# Facile Fabrication of Highly Active Magnetic Aminoclay Supported Palladium Nanoparticles for the Room Temperature Catalytic Reduction of Nitrophenol and Nitroanilines

**DOI:** 10.3390/nano8060409

**Published:** 2018-06-06

**Authors:** Lei Jia, Wensheng Zhang, Jun Xu, Jianliang Cao, Zhouqing Xu, Yan Wang

**Affiliations:** 1 School of Chemistry and Chemical Engineering, Henan Polytechnic University, Jiaozuo 454000, China; jlxj@hpu.edu.cn (L.J.); zhangwenshenghpu@163.com (W.Z.); caojianliang@hpu.edu.cn (J.C.); zhqxu@hpu.edu.cn (Z.X.); 2 School of Safety Science and Engineering, State Key Laboratory Cultivation Base for Gas Geology and Gas Control, Henan Polytechnic University, Jiaozuo 454000, China

**Keywords:** Pd nanoparticles, aminoclay, magnetic, 4-nitrophenol, nitrophenol and nitroanilines

## Abstract

Magnetically recyclable nanocatalysts with excellent performance are urgent need in heterogeneous catalysis, due to their magnetic nature, which allows for convenient and efficient separation with the help of an external magnetic field. In this research, we developed a simple and rapid method to fabricate a magnetic aminoclay (AC) based an AC@Fe_3_O_4_@Pd nanocatalyst by depositing palladium nanoparticles (Pd NPs) on the surface of the magnetic aminoclay nanocomposite. The microstructure and the magnetic properties of as-prepared AC@Fe_3_O_4_@Pd were tested using transmission electron microscopy (TEM), energy-dispersive X-ray spectroscopy (EDS), X-ray diffraction (XRD), and vibrating sample magnetometry (VSM) analyses. The resultant AC@Fe_3_O_4_@Pd nanocatalyst with the magnetic Fe-based inner shell, catalytically activate the outer noble metal shell, which when combined with ultrafine Pd NPs, synergistically enhanced the catalytic activity and recyclability in organocatalysis. As the aminoclay displayed good water dispersibility, the nanocatalyst indicated satisfactory catalytic performance in the reaction of reducing nitrophenol and nitroanilines to the corresponding aminobenzene derivatives. Meanwhile, the AC@Fe_3_O_4_@Pd nanocatalyst exhibited excellent reusability, while still maintaining good activity after several catalytic cycles.

## 1. Introduction

Recently, noble metal nanocatalysts have received much attention due to their high catalytic efficiency in many catalytic reactions including hydrogenation, dehydrogenation, oxidation, and so on [1,2,3,4,5]. However, noble metals are expensive and scarce resources, which severely limits their large-scale use. Therefore, it is important to increase the utilization rate of noble metals. The catalytic activity of the noble metal nanocatalyst is responsible for the catalytic reaction. It is reported that only the first layer of metal material responsible for the catalytic reaction. Therefore, in order to improve the specific surface area, it is necessary to synthesize metal nanoparticles that have small size and high dispersion [6]. However, as the diameter of the noble metal nanoparticles decreases, two problems arise: firstly, the surface energy gradually increases, which leads to the aggregation of the noble metal nanoparticles; secondly, the nanoparticles are difficult to separate from the reaction solution. These disadvantages generally result in reduced catalytic activity and reusability. In order to overcome the above drawbacks, loading nanoparticles onto a suitable carrier is a viable solution [1]. Therefore, noble metal catalysts were often loaded onto various solid supports such as silica [7,8], silicon nanowires [9,10], magnetic microspheres [11,12], carbon-based materials [13], or metal oxides [14,15,16]. Among the magnetic catalyst supports, Fe_3_O_4_ is an ideal carrier, which is easy to prepare and has a very active surface for adsorbing/immobilizing metals and ligands. It can not only prevent aggregation of metal nanoparticles, but also promote recirculation of nanocatalysts by magnetic separation [4,5].

Among various noble metal nanoparticles, Pd nanoparticles (Pd NPs) have extremely small size and high surface-to-volume ratios and have received great attention in the past decades [6,16]. Pd NPs have demonstrated outstanding effectiveness as catalysts for catalytic properties in different organic reactions on chemical and pharmaceutical industries, including hydrogenation and C–C coupling reactions [17,18]. For example, Naghipour et al. [19] reported that Fe_3_O_4_@CS-Schiff based Pd nanocatalysts showed higher catalytic activity for Suzuki-Miyaura and Heck-Mizoroki C–C coupling reactions. Molga et al. [20] reported a palladium on alumina catalyst as being a highly selective homogeneous catalyst for the reduction of 2,4-dinitrotoluene.

Since the organo-functionalized clays were first described in 1997 [12], layered clay materials that can be synthesized to tune their surface-charge properties have recently emerged as promising scaffolds for constructing functional materials [21]. As a representative, aminoclay (AC) is a layered clay of organic magnesium silicate, which shows excellent water-solubility as it can be self-exfoliated in water under electrostatic repulsive forces generated from protonated amino-groups [21,22]. Meanwhile, aminoclay is the most representative of the layered clays, its R group is –(CH_2_)_3_NH_2_ and relative molecular mass is 1156 [23], and the distance between the centers of two parallel lamellae is about 1.7 nm. Due to the good water-soluble of amino-clay, the noble metal nanoparticles supported on the surface can be brought into complete contact with the reactants to accelerate the reaction rate.

Based on the above consideration, we synthesized a facile, low-cost, and efficient catalyst AC@Fe_3_O_4_@Pd, with Pd NPs immobilized on the surface of magnetic aminoclay@Fe_3_O_4_ (AC@Fe_3_O_4_). There was no material loss during the entire synthesis and the yield of the final nanocatalyst was high. AC@Fe_3_O_4_ was prepared through the sol-gel process and solvothermal method. And then, Pd NPs were fabricated on the surface of AC@Fe_3_O_4_ through Pd(NO_3_)_2_ followed by sodium borohydride (NaBH_4_) reduction in water at room temperature. The AC provides a large surface area on which the Pd NPs were finely dispersed. The AC@Fe_3_O_4_@Pd exhibited high catalytic efficiency for the reduction of 4-nitrophenol (4-NP) and various nitroanilines by sodium borohydride. Compared with the aminoclay-metal nanocomposites in the literature [21,24,25], the catalyst we prepared not only has good water dispersibility, but also has magnetic properties. Although the non-magnetic aminoclay-metal complex also has certain catalytic activity [26,27,28], it is difficult to achieve rapid separation of the catalyst from the substrate because of its excellent water solubility after the reaction is completed, which affects the efficiency of recycling. Furthermore, the catalyst could be easily separated from reaction solution by applying external magnetic fields for several cycles with good recycling stability in reaction mixture.

## 2. Results and Discussion

### 2.1. Characterization of AC@Fe_3_O_4_@Pd

**Figure 1:**
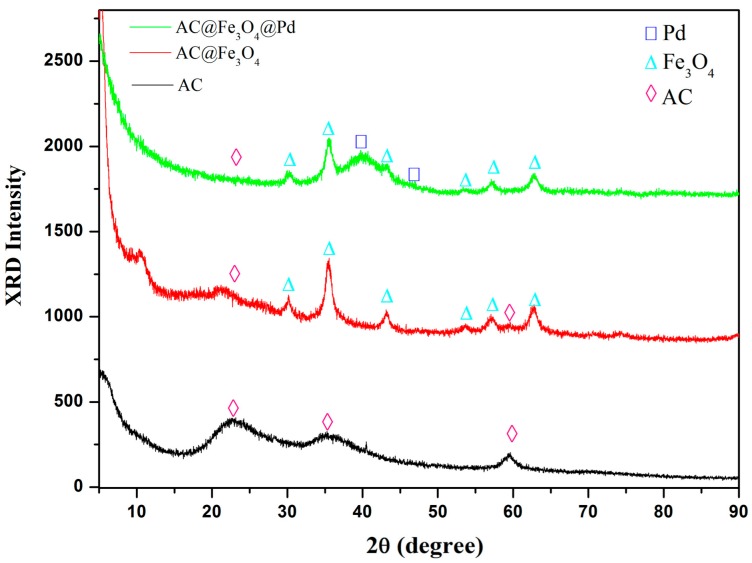
XRD patterns of AC, AC@Fe_3_O_4_ and AC@Fe_3_O_4_@Pd.

The crystalline structure of the as-obtained sample was investigated by XRD analysis. Figure 1 displays the XRD patterns of AC, AC@Fe_3_O_4,_ and AC@Fe_3_O_4_@Pd. The original aminoclay (AC) exhibits four peaks centered at 2θ = 22.9°, 34.8°, and 59.6°, which was in agreement with the literatures [21]. The diffraction patterns for the magnetic AC@Fe_3_O_4_ displayed six characteristic peaks at 30.2°, 35.5°, 43.1°, 53.4°, 57.1°, and 62.8°, corresponding to the (220), (311), (400), (422), (511), and (440) planes of the face centered cubic (fcc) structures of the Fe_3_O_4_ nanoparticles (JCPDS No. 75-1609), respectively. While after loading the Pd nanoparticles, the XRD pattern of the AC@Fe_3_O_4_@Pd presented almost the same feature as those shown in AC@Fe_3_O_4_, besides a broad peak at 2θ = 40.1°, which corresponded to the amorphous peak of Pd(111) diffractions in Pd *fcc* crystals. This clearly demonstrated that the well-crystallized Pd NPs were loaded on the surface of magnetic AC@Fe_3_O_4_.

**Figure 2:**
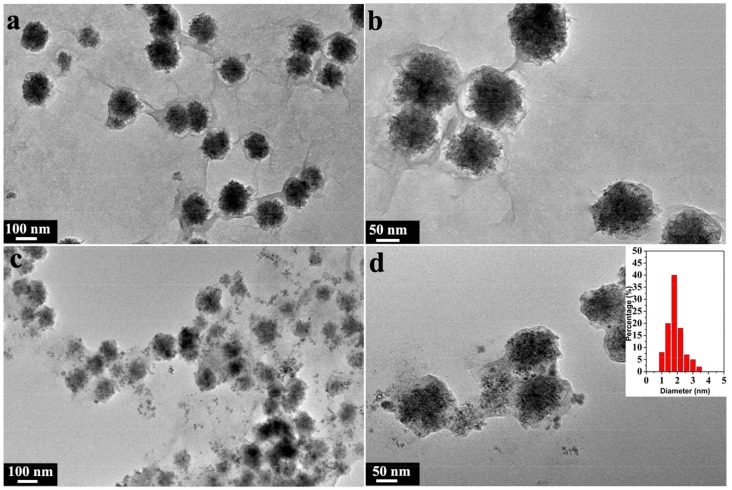
The TEM images of the magnetic AC@Fe_3_O_4_ catalyst carrier (**a**, **b**) and the final AC@Fe_3_O_4_@Pd catalysts (**c**, **d**).

For the prepared Mg-aminoclay, it indicated excellent water-soluble ability by delaminated clay sheets, which is associated with protonated amine (–NH_3_^+^) formation with a high density. Figure 2 shows the transmission electron microscopy (TEM) images of the magnetic AC@Fe_3_O_4_ and AC@Fe_3_O_4_@Pd, which indicated that the aminoclay still maintained the sheet-like morphology, which was according to the previously reported microphotographs [29,30,31]. What was different from the original aminoclay was that, there were some more uniformly distributed Fe_3_O_4_ nanoparticles on the surface of the sheet-like structure, which formed a core-shell nanocomposite (Figure 2a,b) and lain the foundation of the catalyst reuse through the simple magnetic separation. Finally, a layer of Pd nanoparticles on the above AC@Fe_3_O_4_ catalyst carrier was fabricated by the classical reduction method, as shown in Figure 2c,d.

**Figure 3:**
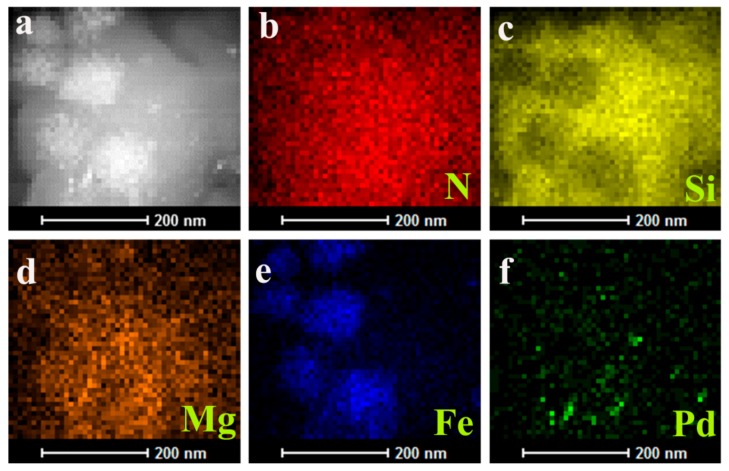
High angle annular dark-field scanning transmission electron microscopy (HAADF-STEM) images of AC@Fe_3_O_4_@Pd (**a**); energy-dispersive X-ray spectroscopy (EDX) mapping of N element (**b**); Si element (**c**); Mg element (**d**); Fe element (**e**) and Pd element (**f**).

**Figure 4:**
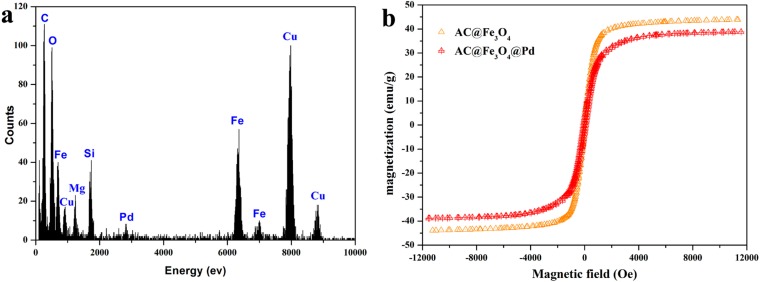
(**a**) EDX spectrum of AC@Fe_3_O_4_@Pd nanocatalysts. (**b**) Room-temperature magnetization hysteresis loops of the as-prepared AC@Fe_3_O_4_ and AC@Fe_3_O_4_@Pd.

The chemical composition and elemental distribution of AC@Fe_3_O_4_@Pd were investigated by EDX-mapping and the energy-dispersive X-ray spectroscopy (EDX). Clearly form Figure 3, N, Si, Mg, Fe, and Pd elements were distributed on AC@Fe_3_O_4_@Pd. The zone of the distribution of N, Si, and Mg elements was larger than that of Pd and Fe, which was consistent with the result that core-shell structure of the AC@Fe_3_O_4_@Pd was formed. The dark-field images indicated abundant deposition of Pd nanoparticles on the magnetic carrier. Meanwhile, these results also indicated that the Pd elements were evenly dispersed among the magnetic carrier. The EDX spectrum of the AC@Fe_3_O_4_@Pd (Figure 4a) exhibited the presence of N, Si, Mg, Fe, and Pd elements, which also proved the successful deposition of Pd on the AC@Fe_3_O_4_ catalysts carrier. ICP-AES was used to determine the Pd content (about 3.6 wt %) on fresh AC@Fe_3_O_4_@Pd, which was consistent with the designed ratio.

The magnetic properties of the as-prepared AC@Fe_3_O_4_@Pd nanocatalyst and the original catalyst carrier were investigated. Figure 4b shows the M-H hysteresis loops of the AC@Fe_3_O_4_ and AC@Fe_3_O_4_@Pd measured by sweeping the external field between −1.2 and 1.2 T at room temperature. There were no remanence and coercivity on the two magnetization curves, which suggested the AC@Fe_3_O_4_@Pd nanocatalyst still maintained the superparamagnetic behavior after the surface modification. The magnetization value of AC@Fe_3_O_4_@Pd (39.2 emu g^−1^) was lower than AC@Fe_3_O_4_ (44.6 emu g^−1^), which can be due to the loading of non-magnetic Pd nanoparticles. However, the magnetic sensitivity of the as-prepared AC@Fe_3_O_4_@Pd was strong enough to achieve magnetic separation of the nanocatalyst after the catalytic reaction. All the above results reveal that the recyclable magnetic AC@Fe_3_O_4_@Pd nanocatalyst had been successfully fabricated.

### 2.2. Catalytic Activity

**Figure 5:**
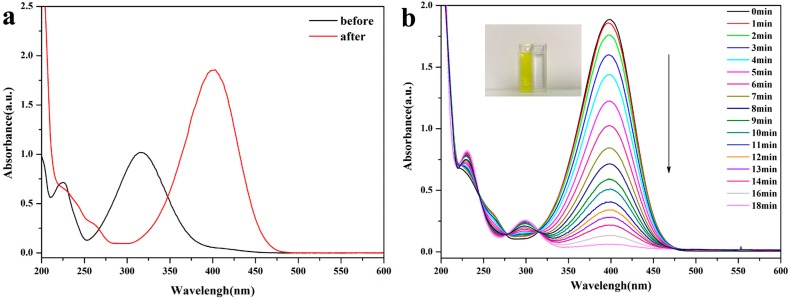
(**a**) UV-vis spectra of 4-NP before and after adding NaBH_4_ solution. (**b**) Absorption spectra observed at different reaction time indicating the decrease in the absorbance intensity at 400 nm and the appearance of a new band at 233 nm and 300 nm.

The reduction of 4-nitrophenol to 4-aminophenol under the action of excess sodium borohydride (NaBH_4_) was carried out to evaluate the catalytic performance of AC@Fe_3_O_4_@Pd nanocatalyst. To start with, 4-NP was added into deionized water; after sonication for about 15 min, the color of solution was light yellow and the UV−vis spectrum showed an absorption peak centered at 318 nm. After the addition of excess NaBH_4_ (1.2 M, 0.25 mL), the color of the above mixed solution changed from light yellow to dark yellow and the UV-vis absorption spectrum of the 4-nitrophenolate salt exhibited a characteristic peak produced by the nitro compound at about 400 nm (Figure 5a) [32,33].

In the absence of AC@Fe_3_O_4_@Pd, there was only a small amount of bubbles without the addition of as-prepared nanocatalyst, due to the slow hydrogen generation between NaBH_4_ and water molecules [34]. However, in the presence of this noble metal nanocatalyst, a large amount of bubbles appeared along with the gradual fading of the bright yellow solution. As shown in Figure 5b, the color of the above mixed solution was restored to the original colorless within 18 min, demonstrating the completion of this catalytic reaction. The intensity of the characteristic absorption peak of 4-NP from UV-vis spectrum at 400 nm quickly weakened until it disappeared within 18 min along with the appearance of two new adsorption peaks at about 233 and 300 nm, suggesting that all of the 4-NP were reduced to 4-AP [35,36].

**Figure 6:**
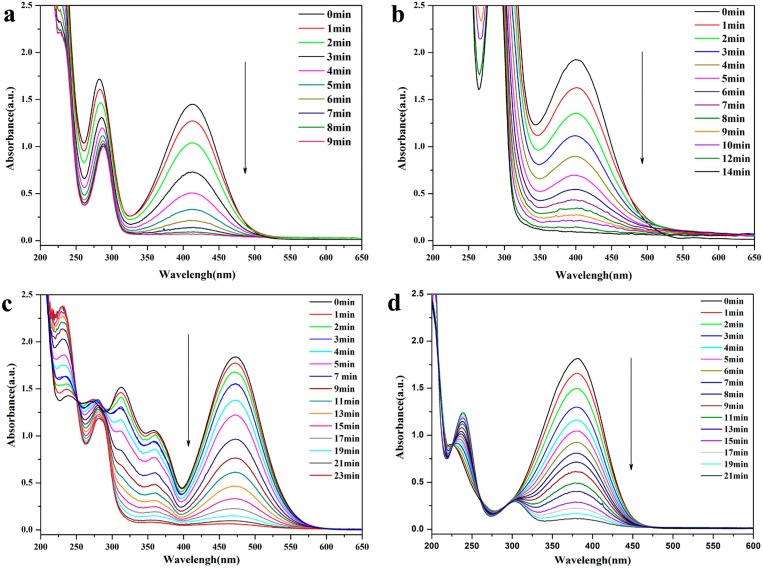
UV-Vis absorption spectra for the reduction of (**a**) o-Nitroaniline; (**b**) m-nitroaniline (**c**) 2,4-nitroaniline; (**d**) p-nitroaniline using AC@Fe_3_O_4_@Pd nanocomposites.

In addition, we also investigated the catalytic properties of AC@Fe_3_O_4_@Pd on the reduction of other nitroanilines such as o-Nitroaniline, m-nitroaniline, p-nitroaniline, and 2,4-nitroaniline. All the catalytic conditions of the above nitroanilines were the same as that of 4-NP and the reaction progresses of these derivatives were monitored by UV–vis spectrometry. As shown in Table 1, the reduction time of each substrate and the conversion were calculated, which indicate that the AC@Fe_3_O_4_@Pd displayed good catalytic performance with satisfactory yield toward nitrophenol and nitroaniline derivatives regardless of different substituent groups. From Figure 6a–d we can also observe that the highest characteristic absorption peak of each substituent gradually weakened within 9–23 min, which proved the excellent percent conversion of the prepared noble metal nanocatalyst.

**Figure 7:**
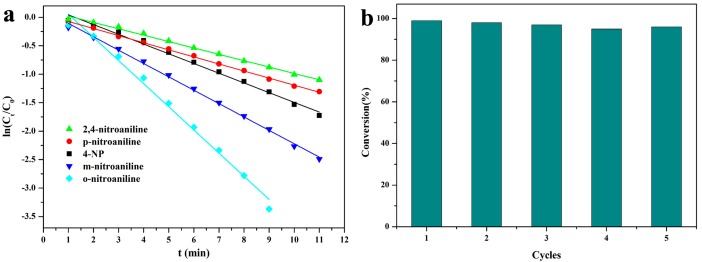
(**a**) Plot of ln(C_t_/C_0_) against time for the reduction of 2,4-nitroaniline (green), p-nitroaniline (red), 4-NP (black), m-nitroaniline (blue), o-Nitroaniline (light green). (**b**) Conversion of 4-NP in successive cycles with AC@Fe_3_O_4_@ Pd.

Since the concentration of NaBH_4_ in the reaction was much higher than the 4-NP concentration, this eliminated the effect of donor BH_4_^−^ on the catalytic reaction. This means that the reaction rate can be considered to depend only on the concentration of 4-NP. Thus, the rate of catalytic reaction was evaluated using pseudo-first-order kinetics, which is given as k_ap_t = ln(C_t_/C_0_), where C_0_ is the initial absorbance of the reagents at the maximum absorption wavelength, C_t_ is the absorbance of reagents at maximum absorption wavelength under different time t, and K_ap_ is the apparent rate constant. In order to quantitatively describe the reaction kinetics, the graphs and reaction times of ln(C_t_/C_0_) are shown in Figure 7a. The reaction rate constant K_ap_ can be calculated from the slope of the linear fit in the Figure 7a, which refers to the rate of degradation of the 4-nitrophenol concentration. The rate constant of our synthesized catalyst was 0.171 min^−1^. It is well known that the rate constant is influenced by many factors, such as metal nanoparticle loading, the usage of NaBH_4_, and catalysts. Table 2 lists some k values and experimental parameters in recent studies. Taking a small usage of catalysts (14 μg) and high k/m_Pd_ value into account, the catalytic performance of AC@Fe_3_O_4_@Pd nanocatalyst was superior than other catalysts based on Pd nanoparticles [37,38,39,40]. Besides the k value, the TOF was also calculated and the value was about 0.928 s^−1^. Compared with recent studies with catalysts based on Pd nanoparticles, the TOF value of AC@Fe_3_O_4_@Pd nanocatalyst was also higher than others [36,41,42].

In addition, the recyclability of a catalyst was very important for the heterogeneous catalytic process [43]. The as-prepared AC@Fe_3_O_4_@Pd nanocatalyst could be easily separated from the solution by using an external magnet, due to its magnetic property. To test the recyclability of the catalysts, five cyclic reactions of the catalyst AC@Fe_3_O_4_@Pd were evaluated. After the hydrogenation of 4-NP was repeated each time, the nanocatalyst was washed and dried and directly used for the next catalysis. As revealed in Figure 7b, with the increase of the number of cycles, the conversion rate slightly fluctuated but little changed, demonstrating the AC@Fe_3_O_4_@Pd nanocatalyst did not lose its initial catalytic activity and still showed good stability and catalytic activity.

## 3. Materials and Methods

### 3.1. Chemicals

Magnesium chloride hexahydrate (MgCl_2_·6H_2_O), 3-Aminopropyltriethoxysilane (APTES), ferric chloride hexahydrate (FeCl_3_·6H_2_O), ethylene glycol, sodium acetate anhydrous, polyethylene imine, palladium nitrate (Pd(NO_3_)_2_), o-Nitroaniline (o-NA), m-nitroaniline, p-nitroaniline, 4-nitrophenol(4-NP), 2,4-nitroaniline, and sodium borohydride (NaBH_4_) were purchased from Sigma-Aldrich Chemical Co. All the chemicals and solvents are reagent grade and received without any further purification. Ultrapure water was prepared by using NANO Pure Infinity System (Barnstead/Thermolyne Corp., Dubuque, Iowa, USA) and was used throughout all the experimental processes.

### 3.2. Preparation of Aminoclay (AC)

AC was prepared by the method according to the previous report [44]: The MgCl_2_·6H_2_O (1.68 g, 8.3 mmol) was dissolved into 50 mL of absolute ethanol under ultrasonic dispersion, APTES (1.68 g, 8.3 mmol) was dropwise added and the solution was stirred at room temperature for 24 h. The light-yellow solid was collected by centrifugation and washed with absolute ethanol for three times. Finally, the obtained solid was dried under vacuum at 80 °C for 6 h.

### 3.3. Preparation of AC@Fe_3_O_4_ Composites

The AC@Fe_3_O_4_ composites were prepared by the method of solvothermal reaction: One gram FeCl_3_ 6H_2_O was dissolved in 30 mL of ethylene glycol to form a clear yellow solution after adding 2.7 g sodium acetate anhydrous and 0.75 g polyethylene imine, the mixed solution was stirred for 30 min. 0.3 g of AC powder was added into the above solution and were fully ultrasound for 3 h. The mixed solution was then poured into a teflon-lined stainless-steel autoclave and reacted at 200 °C for 8 h. After the reaction was completed, the solution was cooled to room temperature and the product was collected with the help of a magnet file. The products were washed with ethanol and deionized water for several times and dried under vacuum at 50 °C for 3 h for further use.

### 3.4. Preparation of AC@Fe_3_O_4_@Pd Composites

The AC@Fe_3_O_4_@Pd were prepared by the method of conventional impregnation and subsequent reduction steps: Pd (NO_3_)_2_ (12 mg, 46.3 mol Pd) and AC@Fe_3_O_4_ (140 mg) were dispersed in 15 mL aqueous solution and the mixture was stirred for 3 h at room temprature. Subsequently, 1.0 mL NaBH_4_ (56 mg, 1.4 mmol) aqueous solution was added to the above solution and the solution was stirred for 1 h under room temperature. Finally, the as-obtained AC@Fe_3_O_4_@Pd composites were separated and collected with a magnet, followed by washing with deionized water three times, and drying under vacuum at 50 °C for 3 h.

### 3.5. Characterizations

Transmission electron microscopy (TEM), high resolution transmission electron microscopy (HRTEM), and the energy dispersive spectra (EDS) were determined using a Tecnai-G2-F30 (FEI, Eindhoven, The Netherlands) at acceleration voltages of 200 kV. X-ray diffraction (XRD) measurements were carried out on a X’pert PRO X-ray power diffractometer (PANalytical Co.X’pert PRO, Almelo, The Netherlands) using Cu Ka radiation of 1.5406 A (40 kV, 30 mA). Magnetization measurements were performed on a vibrating sample magnetometry (VSM, LAKESHORE-7304, Clark County, NV, USA) at room temperature. The UV measurement was finished on a Shimadzu UV-240 spectrophotometer (Shimadzu Corporation, Kyotp, Japan). Pd contents of the samples were determined by inductively coupled plasma-atomic emission spectroscopy (ICP-AES) using an IRIS Advantage ER/S spectrophotometer (ICPE-9800, Shimadzu Corporation, Japan).

### 3.6. Catalytic Studies

The catalytic property of the as-obtained AC@Fe_3_O_4_@Pd for the reduction of nitrophenol and nitroanilines: In a typical reaction, amounts of 8 mL of deionized water was mixed with 0.25 mL (3.4 × 10^−3^ M) of 4-nitrophenol and 0.25 mL of NaBH_4_ (1.2 M). After forming a homogeneous dispersion, 3 mL of the above mixture was transferred into quartz cuvette in sequence. Immediately after adding 14 μL of 1 mg/mL AC@Fe_3_O_4_@Pd, it could be observed that the solution color changed gradually vanished as the reaction proceeded. The reaction progress was quickly measured by UV-vis spectroscopy in a scanning range of 200–600 nm under the speed of 1200 nm/min (UV-240, Shimadzu Corporation, Japan). The yellow color of the solution gradually vanished, indicating the formation of 4-aminophenol. Following the similar procedures, AC@Fe_3_O_4_@Pd was also used as catalyst for the reduction of other nitroanilines. In the recycling study, the AC@Fe_3_O_4_@Pd nanocatalyst was separated from the solution by using magnet when the reduction reaction completely finished. After washing using water for three times, they were reused in the next reaction run. The procedure was repeated ten times.

## 4. Conclusions

In conclusion, the magnetic recoverable AC@Fe_3_O_4_@Pd nanocatalyst was fabricated through a facile way. The good water-solubility of the original aminoclay makes the final catalyst fully dispersed in water, which greatly increased the contact chance between the catalyst and substrates. The catalytic ability of AC@Fe_3_O_4_@Pd was confirmed by the study of the reduction of nitrophenol and nitroanilines to the corresponding aminobenzenes, which indicated the AC@Fe_3_O_4_@Pd had good catalytic performance with satisfactory yield toward nitrophenol and nitroanilines regardless of different substituent groups. Furthermore, the AC@Fe_3_O_4_@Pd can be separated from reaction system conveniently with the help of an external magnetic field, which gave the reusability of the prepared nanocatalyst. The as-obtained AC@Fe_3_O_4_@Pd nanocomposite may become an ideal recyclable catalyst for the reduction of other aromatic compounds owing to its stability, dispersibility, and efficient magnetism.

## Figures and Tables

**Table 1:** Reduction of various nitrophenol and nitroanilines using AC@Fe_3_O_4_@Pd (Reaction condition: 0.25 mL of 3.4 × 10^−3^ M substrates and 0.25 mL of 1.2 M fresh NaBH_4_ at the room temperature; the amount of catalysts dosage is 14 μg in every reaction and the conversion is more than 98%).

Compound	Time/min	Rate Constant/min^−^^1^
p-Nitroaniline	21	0.124
m-Nitroaniline	14	0.235
o-Nitroaniline	9	0.407
2,4-Nitroaniline	23	0.119
p-Nitrolpenol	18	0.171

**Table 2:** Comparison of k for the reduction of 4-NP in different catalytic systems.

Catalysts	Catalysts Dosage (mg)	Pd Loading (wt %)	NaBH_4_ Dosage	K Value (min^−1^)	K/m_Pd_ Value (min^−1^ mg^−1^) ^a^	References
Mesoporous Pd leaves	0.25	Nearly 100	0.3 mL × 0.1 M	0.49	1.96	38
Pd/rGS	1.0	0.5	0.1 mL × 0.01 M	0.141	28.2	39
Pd/rGS	0.5	7.5	1.0 mL × 0.1 M	0.27	7.2	40
Fe_x_O_y_/Pd@mSiO_2_	0.1	1.9	0.5 mL × 0.26 M	0.096	50.53	41
AC@Fe_3_O_4_@Pd	0.014	3.6	0.08 × 1.2 M	0.171	339.29	Here

^a^ The value of k/m_Pd_ was calculated based on the k value and mass of Pd nanoparticles in corresponding catalysts.
